# Gossip

**Published:** 1863-01

**Authors:** 


### Art. XIII.—GOSSIP.

"Of those diverse gifts which, our Apostle Paul saith, God hath bestowed on man, this physick is not the least, and especially conducing to the good of mankind. Next therefore to God, in all our extremities (*for of the Most High cometh healing*, Ecclus. xxxviii. 2) we must seek to, and rely upon the physician, who is *manus Dei* (saith Hierophilus), and to whom He hath given knowledge, that He might be glorified in his wondrous works." So writes old Burton, and this was the chief theme of the Introductory Lectures with which the present medical year was inaugurated, in the different Medical Schools of the Metropolis, the day our last impression issued from the press. No nobler theme could have occupied the lips of the lecturers, or more fittingly and usefully engaged the mind of the student, new or old, and inducted him into the labours of the Session. And yet in the iteration of this theme, year after year, under the same circumstances, some find a sameness even unto weariness. They would divest, in a great measure, these addresses of that ideal portraiture of the dignity of physic and physician, which, however much we strive, is hardly to be realized, and would convert them into lessons on the actual condition of the profession and the practitioner—of the difficulties and hardships which beset both the one and the other, the inappreciation by the public, the contumelious treatment meted out by representative bodies, such as boards of Guardians, the shortcomings of the Medical Corporations, the internal feuds and the external annoyances, from which medicine and its representatives suffer. For our part we infinitely prefer the wholesome sameness of the course commonly pursued. We object entirely to these Introductory Addresses being ever converted into a means of laying before the public an annual statement or a criticism of professional wrongs and grievances, internal and external—for to this the change suggested would come. What kind of an induction would that be which, so far from leading the student first to contemplate medicine in its noblest and purest aspects, would require of him to regard it in its most revolting phases—in the bickerings and squabblings within its ranks, and the petty and irritating injustice outside—phases which can be obliterated only in proportion as the great ideal of the physician, depicted by Burton, is approached. To adopt such a form of induction for the tyro would be most harmful to him, by presenting medicine, when first drawn near to, in the most grovelling point of view, and could be of no value to any one except as a public lifting up of wrongs actual or assumed.

Happily there was little tendency to this course at the opening of the present Session. Mr. Fergusson, at King's College, pitched his note in the very highest key, and withal was thoroughly practical and instructive, doing the amplest justice, not only to his noble calling as a surgeon, but also to the great College he then represented. After speaking of the important part which the doctor performs in averting, by hygienic measures, evils from the community, and illustrating this in the arrangement of a great hospital, he added :—

"It would, however, gentlemen, be a miserable boast on the part of our profession were we to claim honour merely on the ground thus indicated. My impression is that we have higher data upon which to pride ourselves. We claim a prominent recognition amongst those who give progress to civilization. The churchman inculcates, in his holy mission, our lessons from on high ; the statesman gives laws to the social creed which constitutes a main feature of civilization ; the lawyer interprets and gives civil force to the social obligations which man puts upon man ; the warrior cuts, rightly or wrongly, the Gordian knot ; and we, gentlemen, have our work before us—work which, in my humble opinion, may embolden us to claim a position not inferior in natural grandeur to any calling within the circle of civilization. . . . . It is sufficient for my present purpose to state that, in my mind, no argument, no sophistry of modern speculators, breaks the hallowed charm of the first chapter of Genesis. Again I remind you of the simple and impressive words, 'God created man in his own image.'

"Now, gentlemen, it is that 'image' which you have to study. You have as much to do with the intellectual part as any of your fellow-creatures; but it is your special mission to study the wondrous physical structure of this machine, and that marvellous vital principle which makes the machine from God's power so far to excel that emanating from the highest human intellect.

"Hence, then, I say, that our mission is one of the highest on earth when compared with that of each fellow-labourer. Honouring, as we should, the loom, the steam-engine, the locomotive, and all that gives glory to human intellect, we have in ourselves a mechanism and a motive power which it is the solace of our minds to consider as emanating directly and solely from the Deity. To appreciate the works of the Almighty is the highest and best-received proof of our civilization, as well as of our recognition of a Supreme Being, and of a great hereafter."

Equally high was the note struck by Dr. R. Martin, at St. Bartholomew's Hospital. He told the students the story of the lives of some of the men who had contributed to the success of the St. Bartholomew's school of Medicine, indicated the advantages of the collegiate and prize system to the students, and ended by saying that "he had endeavoured to teach them from the example of those good men whom he had commemorated, how industry, perseverance, and good conduct, and a careful using of the talents committed to their care, will safely and surely lead them to the highest position in the profession—a position which the exercise of a true Christian charity will best enable them to grace."

At the Charing-Cross Hospital, Dr. Headland gave a natural history, as it were, of the physician, describing him as the discoverer, teacher, leader, and reformer, criticized the therapeutical merits and demerits of the day, and cautioned the students that: —" The rewards which the physician might expect for a life of toil and self-devotion were not the highest the world had to bestow. In the esteem of mankind at large, a man who made two men live where but one lived before, was not worthy of any special praise. The improver of the breed of oxen was held in more honour than the improver of the breed of men. The man who had removed the duty from some trivial article of import —he who had been sent as ambassador to conclude a treaty of amity and commerce with the King of the Cannibal Islands— such men received peerages and statues. A man who should rescue our youth from the scourge of consumption, or discover a method of preventing small-pox, might seem to us scarcely less worthy. Yet the pitiful memorial which in the public square hard by had been raised to Jenner had been banished, even with ignominy, from the honourable neighbourhood of men esteemed great because they killed their fellow-creatures, whereas he only saved them. But it did not matter much. A thousand years hence it would be of small importance to us in what estimation we happened to have been held by the world."

At the St. George's Hospital, Mr. Prescott Hewett addressed much solid and instructive advice to the younger students, concerning the method of pursuing the studies they were about to enter upon, pointing his moral with happy illustrations from the lives of Sir Benjamin Brodie and Henry Gray, who were held up as bright exemplars. On one important subject he remarked:—" Leisure hours the student must and ought to have, and it was a question of vital importance how these leisure hours were to be spent. If spent in vice or in idleness, the whole life might hence get a colouring which it would never lose. But students about to enter the profession were fortunate, inasmuch as a good preliminary education was nowadays demanded of them, and men who once had had given to them the rudiments of a good education could never be at loss how to spend any leisure time they might have. The medical man might in his professional avocation be called upon to mix with the best and the most highly educated society, and if he had, unfortunately, neglected his general education, would soon find himself at a disadvantage; and bitter would then be his regrets that he had, whilst a student, allowed his leisure hours to pass without turning them to good account. A portion of the student's leisure time was then to be spent, first in acquiring a thorough knowledge of his own language, and then of French and of German."

At the Grosvenor-place School of Medicine, Dr. Cholmeley discoursed largely of the nobility of the medical art, the just way of acquiring that art, and the higher duties of the student. Towards the close of his address he remarked: "But while doing your duty to your profession and to those who may in future be entrusted to your care, by observing with perseverance and pertinacity the course of disease in hospital patients, forget not also the duty you owe to these patients. I hope I need not warn you to treat them with all possible gentleness, delicacy, and courtesy; do you unto them as you would that others should do unto you. As gentlemen you cannot fail in this. But, more, regard them with reverence, with gratitude; they are stricken with poverty as well as disease. From them you will gain the knowledge that will fit you for your profession; to them you will owe the power of doing good to others. Whatever success and distinction you may gain in life, you will owe to the poor fellow-men lying in the long rows of beds, and weariness, pain, and sickness in the hospital wards. Oh, never lounge and loiter in those temples of adversity and disease! enter them not save with fervent desire to learn how to help and comfort the suffering, gratefully and reverently seeking this knowledge from the sufferers there, and they will feel and acknowledge the purpose and hope that conduct and rule you, and will themselves be comforted and consoled thereby."

The loftiness of Mr. Forster's teaching at Guy's Hospital may be judged from the following sentences:—

"He honestly believed that no better preliminary education could be devised for any man than that acquired within an hospital, being as it was a knowledge of man, of the whole world, and of science in its profoundest and its most practical bearings; and the medical man—if really one—being a philosopher, a man of sentiment, of thought, of action—called by his duties for, and furnished by his education with, all the qualifications, resources, and habits which perfect or adorn human nature. . . . . Nor must they imagine that the dissecting-room and the operating-theatre blunted the sensibilities to more than a certain extent. No class were so sensitive to the pain of others, so ready to sacrifice self, to resign comforts and enjoyments for the sake of relieving suffering, as medical men. Again: though the vices and the weaknesses of humanity were open to them more than to any others, ought not the lessons to be derived from the contemplation of these things to be more than compensation for the painful scenes often presented to their view? And not only would they derive, but also confer, great benefits; for medical men were usually the friends, the advisers, the father confessors of their patients, and possessed of greater influence over them than men of any other profession. If they worthily discharged their duties, none more closely walked in the steps of the Great Physician. These, the lecturer observed, were privileges to be aspired to, but not to be purchased without effort; and in the few years of an hospital career, an immense amount of knowledge must be concentrated as preparation for a life of immeasurable value and happiness, or the foundation laid for a life of constant regret."

In what admirable fashion Mr. Hutchinson discoursed at the London Hospital, the following eloquent passage will show:—

"To say that medical life made incessant demands on the exercise of the affections; that it brought men into contact with their fellows under circumstances the most likely to call out 'the charities which soothe, and heal, and bless,' was but to state what every one would acknowledge. In like manner it was not needful to insist that it gave the fullest possible play to the faculties of reason, reflection, observation, and memory. With regard to the imagination, however, there was an impression abroad which he took leave to consider as a great mistake. It was thought that men of science, and physicians especially, ought to repress this, the noblest of our mental endowments. If the truth were so, it would indeed be a humiliating fact; but, so far from it, he would undertake to assert, that in no avocation was a healthy and vigorous power of imagination more useful. Let it be remembered that there is a broad distinction between imagination and fancy. The one deals with the unseen, but real; the other with that which is neither seen nor real. Imagination is, indeed, rightly considered, the art of bringing before the mind's eye a clear image of that which is not really under the sight. There may, of course, be errors of imagination, just as there are errors of reasoning, errors of memory, and errors even of sight itself. Now, as the majority of objects with which we have to deal are not at the time actually under view, the power of forming clear, definite, and truthful images of them becomes of the utmost importance; it is, in fact, the only way in which muddle-headedness can be avoided, and its greater or less development constitutes one grand difference between individual men. To a good surgeon, with a clear image-forming faculty, a man's body becomes transparent. Is a bone dislocated, he sees through skin and muscles right down to the seat of injury, and recognises the exact relative position of the now displaced parts. If it be objected that this is the work rather of memory and of previous training than of imagination proper, the reply must be, that all imagination is necessarily built on memory. It is, however, far more than mere memory, since it can advance forward from the facts furnished by memory to others, which are only to be arrived at by inference. One man is able to form very correct ideas of objects—countries, for instance—which he has never seen; whilst another requires that everything should be subjected to his eyes before he has any definite conception of it. The difference is in the power of imagination. An ingenious writer has remarked, that he who first used the expression *ex pede Herculem* must have been a very stupid man, since it is surely not needful to see a whole foot in order to infer the stature of the man. A hair ought to suffice; and he adds: 'To the mind of an archangel a pebble would be a sufficient datum on which to construct the universe.' Tasks somewhat similar in nature are constantly submitted to the surgeon, and he is every day under the necessity of reasoning from a small fact up to a great conclusion. If he cannot imagine accurately much more than is actually before him, his scope is limited indeed. To obtain a clear mental conception of any one of the processes of disease, requires a very sustained exercise of a trained imagination. Indeed, the importance to the surgeon of a careful cultivation of this faculty could scarcely be overrated. How, indeed, are the brilliant achievements of genius accomplished but by the efforts of an imagination so exalted in grasp, so truthful in vigour, that it seems, to ordinary men, to reason without premisses, and to find out truth by inspiration. The poet applies this faculty to the loftiest themes, the surgeon uses it in a more homely field; but in both it is the same power, and in both it is indispensable. Not only is the surgeon indebted to imagination for great assistance in the practice of his art, but he may also draw from its use the most cogent motives for industry and patience. It brings him into sympathy with his patients. Where the prosaic surgeon sees only a stupid, ill-satisfied grumbler, the imaginative faculty will often enable another to glance into the poor fellow's home, and to recognise the fact that his impatience is solely due to the knowledge that every day of his prolonged incapacity for daily work involves the deprivation of daily necessaries to those whom he most loves. Surely such an insight must be most highly calculated to increase our zeal and add zest to our success."

Dr. Priestley, at the Middlesex Hospital, abounded with excellent advice and suggestions. He aptly told the students that "unless they were duly impressed witn the dignity of medicine, they would be as Bunyan's man with the muck-rake, engrossed by ignoble cares, regardless of the golden crown within their reach." He added, "Better than the occupations of ancient philosophers, whose intellects were exercised in mere unprofitable discussions, their work would consist essentially in doing good—in curing disease, and assuaging the sufferings of their fellow-men."

At St. Thomas's Hospital, Dr. Bristow overflowed with the medical history and present troubles of that, for the time being, ostracised foundation, and while showing the folly of the hygienic vagaries of the Governors' Committee, conveyed much instruction to the students.

At University College Hospital, Dr. Wilson Fox's address was an able and learned commentary on the aphorism from the *Novum Organum*:—"It is not possible to run a course aright when the goal itself has not been rightly placed; and the true and lawful goal of the sciences is none other than this, that human life be endowed with new discoveries and powers." He discussed the question whether students and professors really had this goal in view, and whether the course in which they were proceeding towards it would lead to the end which it was sought to attain.

Finally, at the Westminster Hospital, Dr. Anstie dwelt most excellently on the *practical* part of the students' education, at the same time urging other all-important needs. The necessity of acquiring, in addition to technical knowledge, the elements of a really good general education, was dwelt upon. "No calling," he said, "demands so high a standard of general acquirements in those who follow as it does the profession of Medicine, except, indeed, that of the Church. The power of commanding the sympathy of a number of patients, in different ranks of life, is absolutely necessary to considerable professional success; but it is impossible to do this simply by force of the most amiable disposition, or the most genuine kind-heartedness. There must be the power of *intellectual* communion with many different minds, and this can only be gained by a varied intellectual training. It would be absurd to say that the most diligent study of mere technical matters could atone for deficiency in an acquaintance with such important subjects as mental philosophy, the principal events in the history of our race, and the chief developments of its theology, literature, and fine arts. The neglect of such accomplishments as these would account for very much of the ill success of individual medical men, and the undeservedly low social status of the whole profession. Still more necessary was a good working knowledge of the physical sciences, both to professional efficiency and intellectual status with the public. And in these days of free intercommunication of ideas, it would be wrong to omit from the list of accomplishments which the medical man should cultivate, a fair working knowledge of the French and German languages." Further, he insisted upon the importance of the constant cultivation of the moral qualities of courage, foresight, perseverance, self-denial, and above all truthfulness; gave a slight sketch of the present state of pathology and the tendency of modern English therapeutics; and ended by inculcating, in the words of Tennyson, the need of humility:—

> "Our little systems have their day—
> They have their day, and cease to be;
> They are but broken lights of thee,
> And thou, O Lord, art more than they."

On the 1st October, also, Dr. Parkes, F.R.S., inaugurated the Winter Session of the Army Medical School, Fort Pitt, Chatham, by a weighty address on Military Hygiene; and on the 6th of that month, Professor Simonds opened the Session at the Royal Veterinary College. Professor Simonds's lecture was chiefly remarkable for the just view which he enunciated of the true dignity of veterinary science. He commenced his lecture by a brief detail of the principal facts connected with the late outbreak of sheep-pox in Wiltshire. He then proceeded to consider the influence of like outbreaks of epizöotic disease upon the community. Among other illustrations, he pointed out that it was impossible for a people to lose or undergo a diminution of any staple article of food without suffering from the loss. This might not tell upon them at the moment, but sooner or later it would render its account—weakening the powers of resistance against the insidious onslaught of some deadly epidemic. Hence an outbreak of an epizöotic among cattle or sheep may often make the *differential* element in the general outbreak or not, of epidemic disease. Thus outbreaks of disease, such as that which has occurred in Wiltshire, involve questions of national as well as of individual importance—questions touching not only upon personal losses, but on losses seriously affecting the welfare of the whole population. "It is from this point of view," Professor Simonds said, "that we obtain the most just idea of the true dignity of veterinary science and the right place of the vetenary practitioner in the community. From this point also we obtain the most correct estimate of his duties, for it is upon the progress of veterinary science and the skilful exercise of veteririnary art that a community has to depend for its chief safety and protection from the evils arising from epizöotic diseases. This consideration heightens our responsibility to the utmost in the practice of our profession. Inaccurate appreciation of, or indifference or carelessness to this higher responsibility; an error of judgment; a misapprehension of the true character of the disease with which we have to contend, may be a source of immeasurable public suffering—suffering not the less actual because it may escape immediate observation and detection."

The meeting of the British Association for the Advancement of Science, held in Cambridge at the commencement of the quarter, passed off with somewhat less vigour than customary. The heads of Colleges and University dignities of every grade and description, appeared to do their best, but somehow there was an appreciable deadness. The substantial results of the meeting were not, however, below the mark. Excellent papers abounded in the different sections, and a savage scene of intellectual gladiatorship between Professors Owen and Huxley, and Mr. Glashier journeying above the clouds, imparted a sufficiency of sensational element to the morning meetings of the sections to take away the impression of sameness. Dr. Paget presided over the Physiological section.

A special meeting of the Medical Council was held on the 28th of October. In the new *Pharmacopœia* about to be published, it had been proposed to adopt a new *grain* measure, less by one-eleventh than the old grain. The proposition, however, gave rise to so much dissatisfaction in many quarters, and to apprehensions of serious confusion arising from the change, of so reasonable a character, that a special meeting of the Council was very properly summoned to reconsider the matter. As a result of this meeting, it was determined, by a large majority, that the old grain should not be abandoned; and that the only change to be adopted in the scale of weights, should be discontinuance of the terms "drachm" and "scruple"—grains, ounces, and pounds, being the designations alone to be used from the time of publication of the *Pharmacopœia*.

Deaf to all public and professional protest, the managing Governors of St. Thomas's Hospital have held to their original proposition of removing the hospital beyond the boundaries of the metropolis. So determined in fact have they been not to be turned from the course upon which they had originally started, that had it not been from an oversight in their official proceedings, no way of stopping them would have existed, except invocation of the highest powers of the law or of Parliament. In November the entire medical staff of the hospital, except the resident apothecary, addressed a protest to the Governors on the projected removal of the hospital. From this protest we learn that up to that date *the medical staff of the hospital had never been consulted on the question at issue.* That is to say, the *only* persons who could give a trustworthy and just opinion on the propriety or not of the step proposed, had never been asked by the managing Governors to aid them in coming to a right decision. This proves conclusively, what had been previously assumed, that the whole movement is a mere sanitary lune on the part of several of the Governors, amateur sanitarians; and by the indulgence of this freak the sick poor of London are deprived of three hundred beds throughout the winter. The protest of the medical staff is a most able document, and shows in the clearest and most painful light the utter and unjustifiable folly of the proposition to remove the hospital to a suburban or country site. An aspect of wilful perversity is given to the conduct of the Governors, when it is considered that our greatest sanitary authority, Mr. Simon, the distinguished medical officer to the Privy Council, is one of the staff of the hospital. What reason has failed to effect with the Governors, perhaps ridicule may bring about. Unhappy the subjects of *Punch's* "St. Thomas's Pastoral" (Nov. 29), *apropos* of the removal of the hospital "as proposed by the Grand Committee under the auspices of Messrs. Tite and Baggallay." Who can forget these exquisite lines?—

TITE.

"Happy the patient whom with broken bones
The ready cab hath rattled o'er the stones,
For many a mile beyond the lurid pall
Of London smoke to our salubrious hall!
What though the fracture comminuted grow,
And what was simple first now compound show?
Above the shattered frame, his glazing eye
Will drink the azure of unclouded sky:
And when he's lifted fainting from his seat,
He'll have the buttercups beneath his feet.
Should slight concussion to Death's coma pass,
He'll sleep beneath a sod of growing grass:
Should absorbed poison stomach-pumps defy,
What comfort in a purer air to die!

BAGGALLAY.

"Can o'er-tasked surgeons on the journey dwell,
When with pure oxygen their bosoms swell?
Physicians count the minutes that they lose,
'Mongst hedgerows dressed in all their greenest hues?
Students complain the distance does them wrong,
When skylarks hymn, and redbreasts pipe their song?

TITE.

"What if the parents, children, husbands, wives,
Fond phalanx, that with Sunday now arrives,
To greet the inmates of each crowded ward,
From ready access find themselves debarred?
Let them reflect the objects of their care
Breathe less carbonic acid in their air;
Think, mother, from your child though barred you be,
There's hawthorn in the fields, if it could see—

Think, child, whose heart to greet thy sire has bled,
The lambs sport o'er the sward, beside his bed:
If thou his dying eyelids may'st not close,
Outside the wards the wild-flower freely grows."

Garibaldi's wound has passed from a professional almost to a national question. The surgery of rival kingdoms is to be tested by the results of the diagnosis. Mr. Partridge failed to find the ball in the wound, and thought it was not there; Pirogoff and the Italian surgeons were doubtful; Nelaton, coming upon the scene subsequently to the rest, discovered the offending missile. Shortly after one of the Italian doctors happily extracted it, saving in some degree the character of his brethren; and now Russian and English surgery have sunk in the eyes of a wondering continent—the English being deepest in the descent. The picture is not a very pleasant one of a mob of surgeons poking their fingers into the unhappy hero's wound, at all times, fitting and unfitting, but it need not be painted in darker colours than necessary, by the assumption of ignorance on the part of any of the surgeons. The assumption is not warranted by the facts known. The surgeon who conceives that, had Nelaton been upon the spot at the first, he would then have discovered the ball with a facility as great as he did at the last, must have had a most fortunate experience of gun-shot wounds, and Nelaton himself, we imagine, would be the first to disclaim such an assumption—well knowing what his own fate might be in the very next case of gunshot wound with which he might be connected.

Our readers, who may remember a recent article in this Journal (July, 1862), on the Health of the Royal Navy, will be interested to learn that a series of experiments upon the ventilation of ships is being carried out on board H.M.S. *St. Vincent*, at Portsmouth; and, more important still, that the question of the ventilation of the *Royal Oak*, armour-plated frigate, recently launched, was discussed and decided upon, before she left the stocks.

The difficulty of female physicians has again cropped out, St. Andrews being this time the scene of trouble. A lady cleverly effected a lodgment among the medical students, to the no small discomfiture of the University; but it has been decided that she cannot legally be admitted to the classes.

The alarm created by the appearance of typhus at Preston, early in the quarter, is beginning to subside. There was every reason to dread that the outbreak of the formidable disease, among the famine-stricken population, would assume pestilential dimensions. Up to the present time this has not been the case, thanks, probably, to an increased amount of relief given, and the strenuous efforts made to separate the infected and diminish overcrowding. It would be folly, however, to suffer ourselves to be lulled into a false security. The worst part of the winter has yet to come; the germs of the disease are already in the cotton-districts; and we have, at the best, three months of aggravated distress, if not absolute famine, to contend with. The *Lancet* has set a noble example to the profession in this matter, which, we trust, will be largely followed.

---

